# Exploring common circulating diagnostic biomarkers for sleep disorders and stroke based on machine learning

**DOI:** 10.3389/fneur.2025.1599135

**Published:** 2025-10-30

**Authors:** Hanlin Yu, Zhen Wang, Shuya Cai, Yingle Zhang, Xiangzhe Liu

**Affiliations:** ^1^Department of Encephalology, The First Affiliated Hospital of Henan University of Chinese Medicine, Zhengzhou, Henan, China; ^2^Department of General Surgery, The First Affiliated Hospital, Dalian Medical University, Dalian, Liaoning, China; ^3^The Second Affiliated Hospital, Dalian Medical University, Dalian, Liaoning, China

**Keywords:** circulating diagnostic biomarkers, sleep disorders, machine learning, stroke, ARL2

## Abstract

**Background and objectives:**

Sleep disorders (SD) and stroke have long been health concerns. Sleep disorders are known to be a risk factor for stroke, and in recent years it has also been shown that the prevalence of sleep disorders is increased in stroke patients. We inferred that there is some inevitable connection between the two. This study aims to identify common molecular biomarkers and pathways connecting SD and stroke by integrating bioinformatics and machine learning approaches.

**Methods:**

We analyzed transcriptome data from the GEO dataset to identify differentially expressed genes (DEGs). Key biological processes, as well as metabolic pathways, were highlighted by GO and KGEE enrichment analyses. Co-expression modules were then identified in the SD and stroke datasets by weighted gene co-expression network analysis (WGCNA), respectively, and machine learning algorithms (RandomForest, LASSO, and XGBoost) were performed to identify ARL2 as a key diagnostic biomarker with high predictive value (AUC = 0.91). This was finally complemented by animal experiments to verify that ARL2 was upregulated in the experimental group.

**Results:**

In GO and KEGG enrichment analyses, key biological processes such as ‘response to external stimuli’ and ‘organic metabolic processes’ as well as metabolic pathways such as ‘propionate metabolism’ and ‘oxidative phosphorylation’ were significantly enriched, suggesting their potential roles in the pathogenesis of the two disorders. With WGCNA and machine-learning algorithms analyses, we found that ARL2 is an important common marker for both diseases.

**Discussion:**

This study provides insights into the common molecular mechanisms of SD and stroke, highlighting the potential of ARL2 as a diagnostic marker and therapeutic target. Unlike previous studies, we used circulating markers rather than tissue markers, improving the clinical translation in terms of non-invasive, rapid identification of patients at risk for sleep disorders. We need to further investigate the functional role of these genes and their potential in developing targeted therapies for SD and stroke patients.

## 1 Introduction

Sleep disorders (SD) are chronic, recurrent disorders characterized by disturbances in the sleep cycle. According to ICSD-3, SD is classified into seven categories: insomnia, sleep-related breathing disorders, central sleepiness disorders, circadian rhythm sleep–wake disorders, sleep abnormalities, sleep-related movement disorders, and other sleep disorders (1). The most common clinical manifestations of sleep disorders include difficulty falling asleep, incomplete sleep, and excessive daytime sleepiness (2). At the same time, sleep disorders not only reduce quality of life and productivity, but also increase medical and psychiatric problems (3). It is considered a risk factor for many diseases, including cardiovascular events, hypertension (4) and type 2 diabetes mellitus (5). Currently, SD is mainly considered to be a combination of mental disorders (6), environmental factors (7), and circadian rhythm disorders (8) that lead to functional abnormalities in areas of the brain such as the anterior cingulate cortex, the amygdala, and the thalamus (9), but the exact pathogenesis remains unclear. In this study, we focused on common circulating diagnostic markers between insomnia and stroke.

Stroke is a clinical syndrome presenting as an acute, focal neurological deficit, usually caused by vascular injury (e.g., infarction or hemorrhage) to the central nervous system, and is divided into two main categories: ischaemic stroke and haemorrhagic stroke (10). Stroke begins at age 35, with an increased incidence of overall stroke, is common in older adults, and can lead to long-term disability or death (11). Risk factors for stroke include modifiable risk factors such as high blood pressure, atherosclerosis, and arrhythmia, and non-modifiable risk factors such as age, gender, race-ethnicity, and genetics (12). The pathogenesis of stroke is complex, involving excitotoxic mechanisms, inflammatory pathways, oxidative damage, ionic imbalances, apoptosis, angiogenesis and neuroprotection. The end result of the ischaemic cascade triggered by acute stroke is neuronal death and irreversible loss of neuronal function (13). Strokes may occur alone or in conjunction with SD (14).

A systematic review and meta-analysis found that insomnia after stroke is extremely common, with approximately 38.2% of stroke survivors suffering from insomnia or insomnia symptoms, and the prevalence is significantly higher than that of the general population (15). In a prospective cohort, insomnia symptoms were associated with a 1.6-fold increased risk of stroke, and short sleep duration (<6 h) further amplified the risk (16). This suggests that insomnia and stroke may share a common pathological mechanism. Although neurological and cerebrovascular disorders are recognized as distinct entities, their overlapping pathophysiology suggests shared pathways and therapeutic strategies. Patients with both SD and stroke have been shown to exhibit altered inflammatory markers, impaired autonomic regulation and disruptions in circadian rhythms (17–19). Research into the relationship between insomnia and stroke is the most common, with the risk of stroke doubling in people with insomnia. In addition to this, other types of sleep disorders have progressively been shown to be risk factors for stroke (20), and they are also consequences of stroke, according to a meta-analysis of sleep quality after stroke, which showed that poor sleep quality affects 53 per cent of stroke patients (21). There is a close and bidirectional interaction between sleep and blood glucose regulation, a relationship regulated by both sleep–wake homeostasis and circadian rhythms. Insufficient sleep interferes with insulin signaling through multiple pathways, including activation of the sympathetic nervous system, elevated nocturnal cortisol and growth hormone levels, and promotion of lipolysis and free fatty acid release. This is not limited to insufficient sleep; the “chronic hyperarousal” state characteristic of insomnia also exerts independent adverse effects on blood glucose metabolism through persistent activation of the hypothalamic–pituitary–adrenal axis and the sympathetic nervous system (22). In addition, insomnia is commonly associated with metabolic syndrome (23). We found that both metabolic syndrome and type 2 diabetes are risk factors for stroke. Among the 2097 subjects in the Framingham Offspring study, a higher incidence of stroke was obtained in patients with metabolic syndrome than in those with diabetes (24). The hypothalamic–pituitary–adrenal (HPA) axis is a key component of the body’s stress response system. Research consistently demonstrates a bidirectional relationship between HPA axis dysregulation and insomnia: chronic stress activates the HPA axis, leading to hyperarousal and disrupting sleep, while insufficient sleep, in turn, exacerbates HPA hyperactivity. A review of 20 studies revealed that patients with insomnia had moderately elevated cortisol levels, and that there was a positive, but non-significant, correlation between the degree of objective sleep deprivation and group differences in cortisol levels (25). A meta-analysis of 23 prospective cohort studies systematically evaluated the bidirectional association between hypertension and insomnia. The results showed a significant bidirectional positive correlation between the two: insomnia increased the risk of hypertension by 11% (OR = 1.11), while hypertension also increased the risk of insomnia by 20% (OR = 1.20) (26). Hypertension is also a risk factor for stroke (27). We hypothesize that there may be a metabolic association between sleep disorders and stroke. Identifying new diagnostic markers and therapeutic targets for these conditions is crucial to improving patient outcomes. Machine learning to recognize GEO large expression profiles is an advanced and reliable method (28). In this study, we used circulating markers rather than tissue markers, which facilitates ease and speed of testing, clinical translatability, and rapid identification of patients at risk for sleep disorders.

## 2 Methods

### 2.1 Bulk transcriptome data preprocessing

Based on the selection strategy of previous literature, we retrieved SD-related datasets and their corresponding transcriptome profiles of control and IS patients from the Gene Expression Omnibus (GEO) database,^1^ which contains messenger RNA (mRNA) expression profiles and clinical information. Four relatively large transcriptome datasets were identified: GSE208668, GSE16561, GSE22255 and GSE98566. For sleep disorders, we obtained RNA expression data from 17 SD patients and 25 healthy controls from the GSE208668 dataset on the GPL10904 platform (29). For stroke, we obtained 39 control samples and 24 ischemic stroke patients from the GSE16561 dataset as a training set. As each analysis is confined to a single dataset, the issue of inter-dataset batch effects is circumvented. For the transcriptome data mentioned above, we performed gene symbol mapping according to the respective platforms (30). Finally, GSE22255 (31) and GSE98566 (32) were used as independent datasets as retrospective validation, respectively. In case of multiple matches, we took the median and the final expression matrix was obtained by normalization using the log2(X + 1) method. During preprocessing, after an initial quality control check, quantile normalization is performed using the “normalizeBetweenArrays” function in the “limma” package. This method adjusts the expression values so that each sample has the same empirical distribution of expression values, effectively reducing the technical differences between sample.

### 2.2 Preselection of diagnostic biomarkers

The limma package was utilized for differential gene expression (DEG) analysis on the GSE208668 and GSE16561 datasets, following the guidelines for RNA sequencing and microarray studies. DEGs were identified using a stringent cutoff criterion of an adjusted *p*-value (Benjamini-Hochberg false discovery rate, FDR) < 0.05 and an absolute log2 fold change (|LogFC|) > 0.5. To identify common signals across datasets, the final list of common DEGs was defined as the intersection of the DEGs from both GSE208668 and GSE16561.

### 2.3 GO and KEGG enrichment analysis

Gene Ontology (GO) and Kyoto Encyclopedia of Genes and Genomes (KEGG) enrichment analyses were conducted for common driver genes using the clusterProfiler package, an R tool for comparing biological themes across gene clusters. GO analysis was employed to annotate the biological processes, molecular functions, and cellular components associated with the genes, while KEGG was used to annotate the gene pathways. Statistical significance for the enrichment analyses was defined as *p* < 0.05.

### 2.4 WGCNA

Weighted gene co-expression network analysis (WGCNA) was further applied to identify gene modules co-expressed in SD and stroke, revealing their possible shared mechanisms in biological processes such as inflammation, oxidative stress and circadian regulation. Within this analysis, gene significance (GS) was defined as the absolute value of the correlation between an individual gene and the trait of interest. Module membership (MM) was defined as the correlation between the gene expression profile and the module eigengene. To identify highly connected and biologically relevant hub genes, we applied thresholds of GS > 0.2 and |MM| > 0.8. The final list of high-confidence candidate genes was obtained by extracting the intersection of common DEGs (from differential expression analysis) and hub genes (from WGCNA analysis). This multi-step filtering approach ensured that the selected genes were both statistically significant and biologically relevant to the trait under study.and then this genes are subjected to machine learning analysis. We will then analyze these genes by machine learning.

### 2.5 Machine learning selection of diagnostic biomarkers

RandomForest, LASSO and Xgboost were utilized as machine learning methods to identify core genes. RandomForest is an integrated learning method that performs classification and regression analysis by constructing multiple decision trees. It can efficiently process high-dimensional data (e.g., gene expression data) and perform feature selection (33). By calculating the feature importance of 37 genes, it can identify those genes that contribute the most to disease correlation. LASSO is a linear regression model that achieves variable selection and model compression by applying L1 regularization to the regression coefficients. LASSO can simplify the model by penalizing unimportant features (by making their coefficients zero) when dealing with a large number of features, and retaining those genes that are most influential genes (34). XGBoost can accurately model gene expression data to identify key genes associated with diseases and construct accurate prediction models based on these genes (35).

### 2.6 Establishment of animal models

We used a controlled experimental setup. We divided 24 rats randomly (*n* = 4) into four groups (*n* = 6 for each group): normal control group (group 1), sleep disorder group (group 2), stroke group (group 3), and group with both sleep disorder and stroke (group 4). All groups were modeled on 10-month-old male rats. The triggers for sleep disorders in Group 2 and Group 4 mice were sleep deprivation, chronic mild stress, or placing the animals in a disturbed light/dark cycle. Sleep deprivation is achieved primarily by a mild stimulus such as a standardized process of hitting the cage, slightly shaking the cage, or disrupting the sleeping nest when this is not enough to keep the animal awake (36). Middle cerebral artery occlusion (MCAO) was performed on Group 3 and Group 4 rats to simulate stroke. The occlusion model of the middle cerebral artery strictly adopts a permanent MCAO model, induced by intraluminal filament method, i.e., anesthetized rats with chloral hydrous (400 mg/kg, ip) and 3–0 nylon sutures, whose tip is heated near the flame that advances from the external carotid artery to the internal carotid artery, making its tip round until it blocks the origin of the middle cerebral artery (MCA) and leaves the surgical filaments *in situ* 24 mice. Transcranial laser Doppler blood flow method (PeriFlux 5,000; Perimed AB). Blood flow drops to 80% of baseline, indicating successful occlusion of the middle cerebral artery (37). No sleep disturbance or stroke was induced in the control mice. Meanwhile, the feeding and living environments of the 4 groups of rats should be consistent. 4 groups of rats were placed in a comfortable environment with a 12-h light–dark cycle and unrestricted access to water and food. All rats were fed a standard pellet diet, and the amount of food consumed per day was consistent across all groups (38). This experiment passed the ethical review of animal experiments in the First Affiliated Hospital of Henan University of Traditional Chinese Medicine (YFYDW2019035).

### 2.7 RT-qPCR

Total RNA was extracted from the cortex of four groups of mice using the TransZol Up Plus RNA kit (TransGEN, Beijing, China) (38). RNA concentration and quality were then assessed using a nanodrop spectrophotometer (Termo Scientifc, Waltham, MA, USA). Subsequently, reverse transcription was performed using TransScript®OneStep gDNA Removal and cDNA Synthesis SuperMix (AT311, TransGEN, Beijing, China). Amplification was monitored using ChamQ Universal SYBR qPCR Master Mix (Novozymes Q711) and QuantStudio™5 Real-Time PCR System (Thermo Fisher Scientific). Prior to the start of the experiment, we evaluated the expression stability of the candidate internal reference gene *β*-actin in different treatment groups (control, SD, Stroke, SD + Stroke) using the geNorm algorithm. geNorm analysis identifies the most stable internal reference gene by calculating the gene expression stability metric value, M. A lower M value indicates a more stable gene expression. The results of the analysis showed that *β*-actin, with an M value of less than 0.5 (0.335), exhibited a high degree of stability under all experimental conditions and was therefore selected as the data-normalized endogenous gene for this study. Relative gene expression was then determined using the 2^(–ΔΔCT) method. We assessed the statistical significance of the differences in ARL2 expression between the four experimental groups (Control, SD, Stroke, SD + Stroke) using one-way ANOVA analysis. And Tukey’s HSD (Honest Significant Difference) *post hoc* test was used for two-by-two comparison of means between groups. Raw Ct values for each group with their mean, standard deviation (SD) and more specific information are detailed in Supplementary Table 2. Detailed primer sequences are shown in Table 1.

**Table 1:** Primer sequences used for RT-qPCR.

Gene	Primer sequence 5′-3’ Forward	Reverse
ARL2	CAGTCTGGCAGAGAACTGG	GTCAGAGGGAGTGAGAGGA

### 2.8 Independent clinical validation cohort

#### 2.8.1 Data source and study participants

To confirm the clinical relevance of our findings, we conducted an independent retrospective cohort study, which was approved by the Ethics Committee of The First Affiliated Hospital of Henan University of Chinese Medicine (Approval No. 2025HL-389).

We retrospectively enrolled 72 participants admitted between 2024 and 2025, dividing them into four groups of 18 each. The Control group included healthy individuals with no history of stroke or sleep disorders, confirmed by a Pittsburgh Sleep Quality Index (PSQI) score below 5. The Stroke group comprised patients diagnosed with acute ischemic stroke through neuroimaging (CT or MRI), without significant sleep disorder (PSQI score < 7). The Sleep Disorder (SD) group consisted of individuals without a diagnosis of stroke but with significant sleep disturbances, as defined by a PSQI global score greater than 10. Lastly, the SD + Stroke group included patients who met the criteria for both conditions. The Chinese version of the PSQI has been validated and shown to have good reliability and validity in the Chinese population (39).

All participants provided written informed consent upon admission for biospecimen banking, allowing the use of their anonymized clinical data and residual samples for future research. Personal identifiers were removed to protect privacy.

Inclusion criteria for patients were: (1) age ≥ 40 years; (2) hospital admission and collection of baseline blood samples within 24 h of stroke onset or initial clinical assessment; and (3) availability of complete PSQI assessment data. Exclusion criteria were: (1) severe aphasia, impaired consciousness, or cognitive dysfunction (MMSE score < 10); (2) comorbid severe hepatic or renal insufficiency or advanced malignant tumors; and (3) incomplete clinical or biospecimen data.

#### 2.8.2 Experimental procedures: RNA extraction, qRT-PCR and statistical analysis

Peripheral blood samples were collected from all participants using PAXgene Blood RNA tubes. Total RNA was extracted using the PAXgene Blood RNA Kit (Qiagen, Germany) according to the manufacturer’s instructions. RNA concentration and purity were assessed using a NanoDrop spectrophotometer. GAPDH was chosen as the reference gene due to its well-documented stability in human blood transcriptomics studies (geNorm analysis yielded an M-value of less than 0.5 (0.330)). Its stable expression across all groups was confirmed by one-way ANOVA, which showed no significant differences in Cq values (*p* > 0.05). Reverse transcription was performed using the PrimeScript RT Reagent Kit (TaKaRa, Japan). The relative expression levels of ARL2 mRNA were quantified by qRT-PCR using the SYBR Green Premix Pro Taq HS qPCR Kit (Accurate Biology, China), with GAPDH as the internal control. Relative expression was calculated using the 2^(−ΔΔCt) method.

Statistical comparisons of ARL2 expression levels among the four groups were performed using one-way analysis of variance (ANOVA), followed by Tukey’s *post hoc* test for pairwise comparisons. Data are presented as mean ± standard deviation. A *p*-value < 0.05 was considered statistically significant.

## 3 Results

### 3.1 An integrative workflow for identifying common circulatory biomarkers

**Figure 1:**
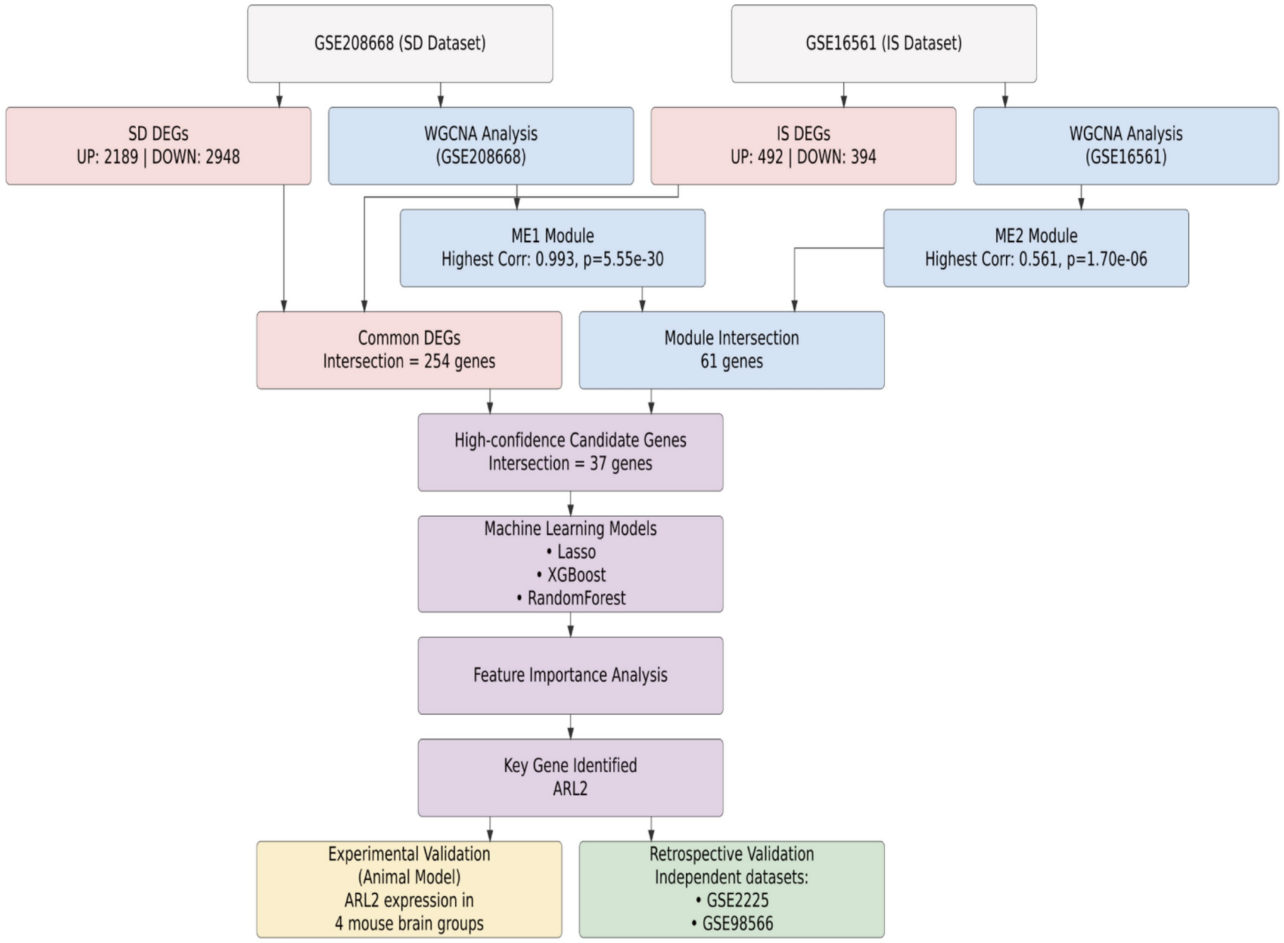
Workflow for the identification and validation of ARL2 as a common diagnostic biomarker. Schematic overview of the multi-step process, integrating transcriptomic data from sleep disorder (GSE208668) and ischemic stroke (GSE16561) datasets, machine learning, and *in vivo* and clinical validation to nominate ARL2 as a shared circulatory biomarker.

To identify circulating biomarkers common to both sleep disorders and ischemic stroke (IS), we implemented a multi-stage discovery and validation pipeline, summarized in Figure 1. Our strategy was predicated on the hypothesis that shared pathological mechanisms between SD and IS would be reflected by common alterations in gene expression. We first defined distinct gene signatures from SD (GSE208668) and IS (GSE16561) transcriptomic datasets independently through differential expression analysis and weighted gene co-expression network analysis (WGCNA). We then integrated these signatures using a consensus approach, prioritizing genes at their intersection. This candidate set was further refined using machine learning feature selection, which converged on ARL2. Subsequently, the diagnostic relevance and specificity of ARL2 were further evaluated through experimental validation of animal models and independent validation datasets (GSE2225, GSE98566).

### 3.2 Identification of differentially expressed genes in SD and stroke

Based on the SD dataset (GSE208668), a total of 5,137 differential genes were identified, and the volcano plot showed that the identified differential genes included 2,948 down-regulated and 2,189 up-regulated genes. In addition, based on the stroke dataset (GSE16561), a total of 886 differential genes were identified, including a total of 394 down-regulated and 492 up-regulated genes. The heat map showed the top 50 most significantly up- and down-regulated genes (Figures 1A,B). Finally, a total of 254 overlapping deg. were created in the SD and stroke datasets.

### 3.3 GO and KEGG enrichment analysis of genes

**Figure 2:**
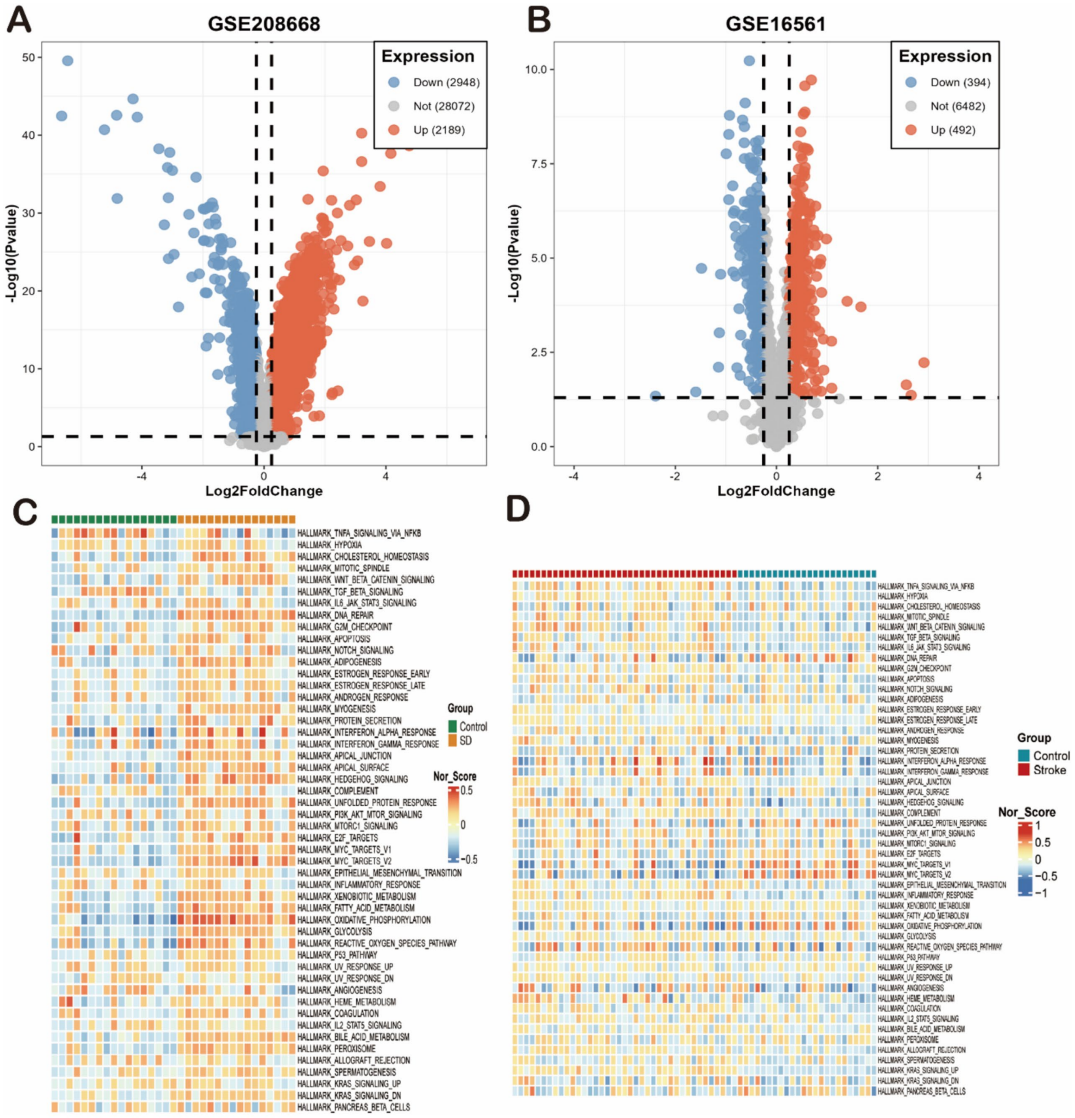
Differential gene expression and pathway enrichment overview in SD and IS models. **(A,B)** Volcano plots of differentially expressed genes (DEGs) from the **(A)** GSE208668 (SD) and **(B)** GSE16561 (IS) datasets. Significantly up-regulated (red) and down-regulated (blue) genes are shown (thresholds: |log2FC| > 0.5, *p* < 0.05). **(C,D)** Heatmaps of enriched Hallmark pathways for the **(C)** GSE208668 (SD) and **(D)** GSE16561 (IS) datasets. Each row represents a gene set, and each column represents a sample or group. Color intensity reflects the normalized enrichment score (NES) or activity of the pathway (red: activated; blue: suppressed).

The SD and stroke modules share 121 overlapping genes, while the DEG module contains 254 shared genes. Given that the modules identified through WGCNA represent groups of genes with similar expression patterns, they may not encompass the full spectrum of DEG genes. In fact, these DEG genes may even diverge significantly from those critical for disease progression. To prevent potential omissions, we integrated DEG genes with the module genes for a more comprehensive analysis (Figure 2).

**Figure 3:**
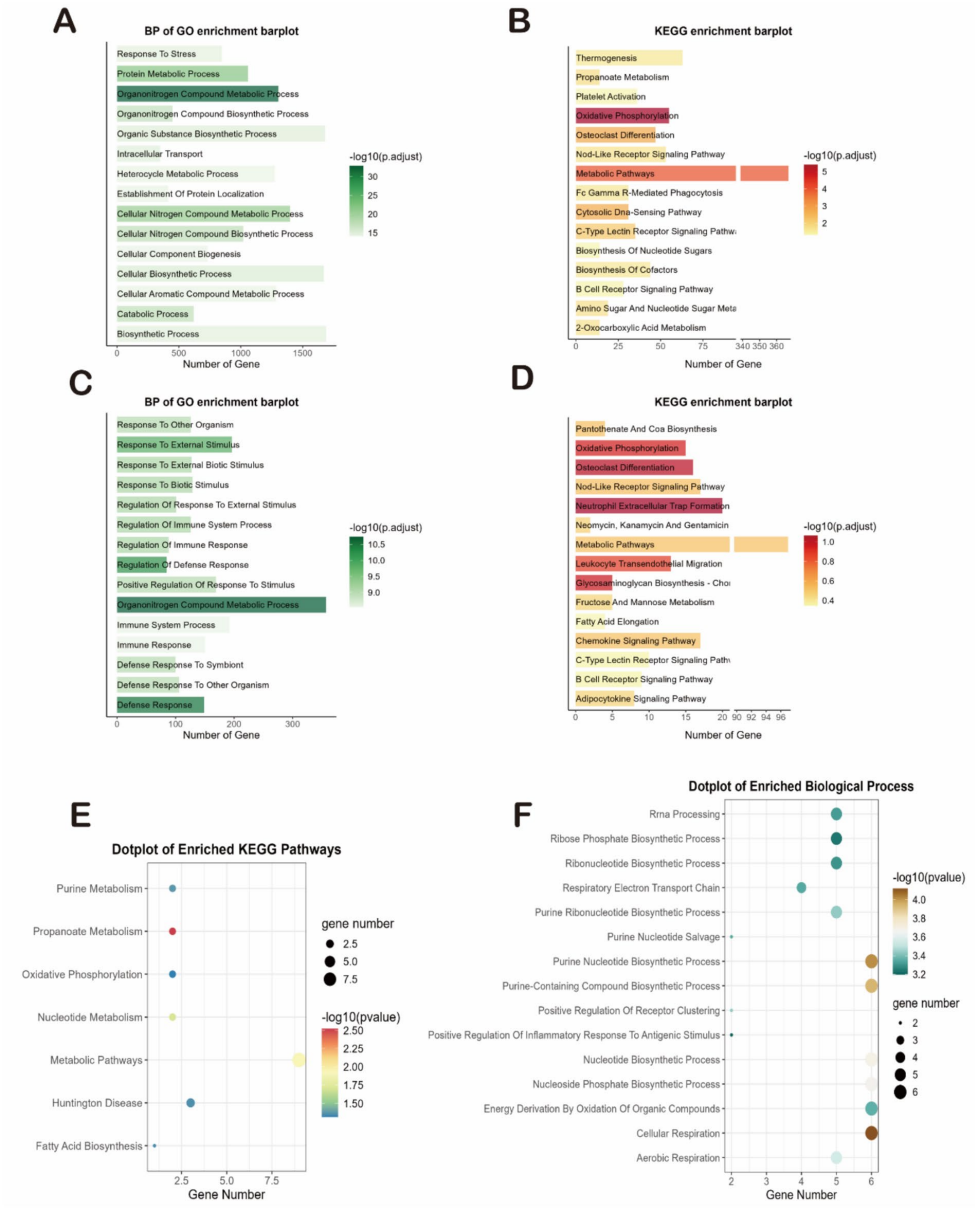
Functional characterization and coregulated pathway analysis. **(A–D)** Bar plots showing the top significantly enriched terms for the DEGs of each dataset. **(A,B)** GO biological processes **(A)** and KEGG pathways **(B)** for the SD dataset. **(C,D)** GO biological processes **(C)** and KEGG pathways **(D)** for the IS datasets. **(E,F)** Enrichment analysis of the 254 common co-driver genes between SD and IS. **(E)** Enriched KEGG pathways. **(F)** Enriched GO biological processes.

We first analyzed these genes for GO and KEGG enrichment. First, The most significantly enriched DEGs in the SD dataset were organitrogen compound metabolic process and oxidative phosphorylation (Figures 3A,B). Secondly, the most significantly enriched DEGs in the IS dataset were organitrogen compound metabolic process, oxidative phosphorylation, neutrophil extracellular trap formation, xidative phosphorylation, defense response, osteoclast differentiation, and leukocyte transendothelial migration (Figures 3C,D). Both diseases had high enrichment of DEGs in the propionate metabolism and redox pathways, so we inferred that the two diseases may be linked in the propionate metabolism and redox pathways. We then performed enrichment analysis on 254 common co-driver genes between SD and IS. We again found that propanoate metabolism and cellular respiration were highly significantly enriched, further validating our hypothesis (Figures 3E,F).

### 3.4 Weighted gene coexpression network analysis of SD and stroke

**Figure 4:**
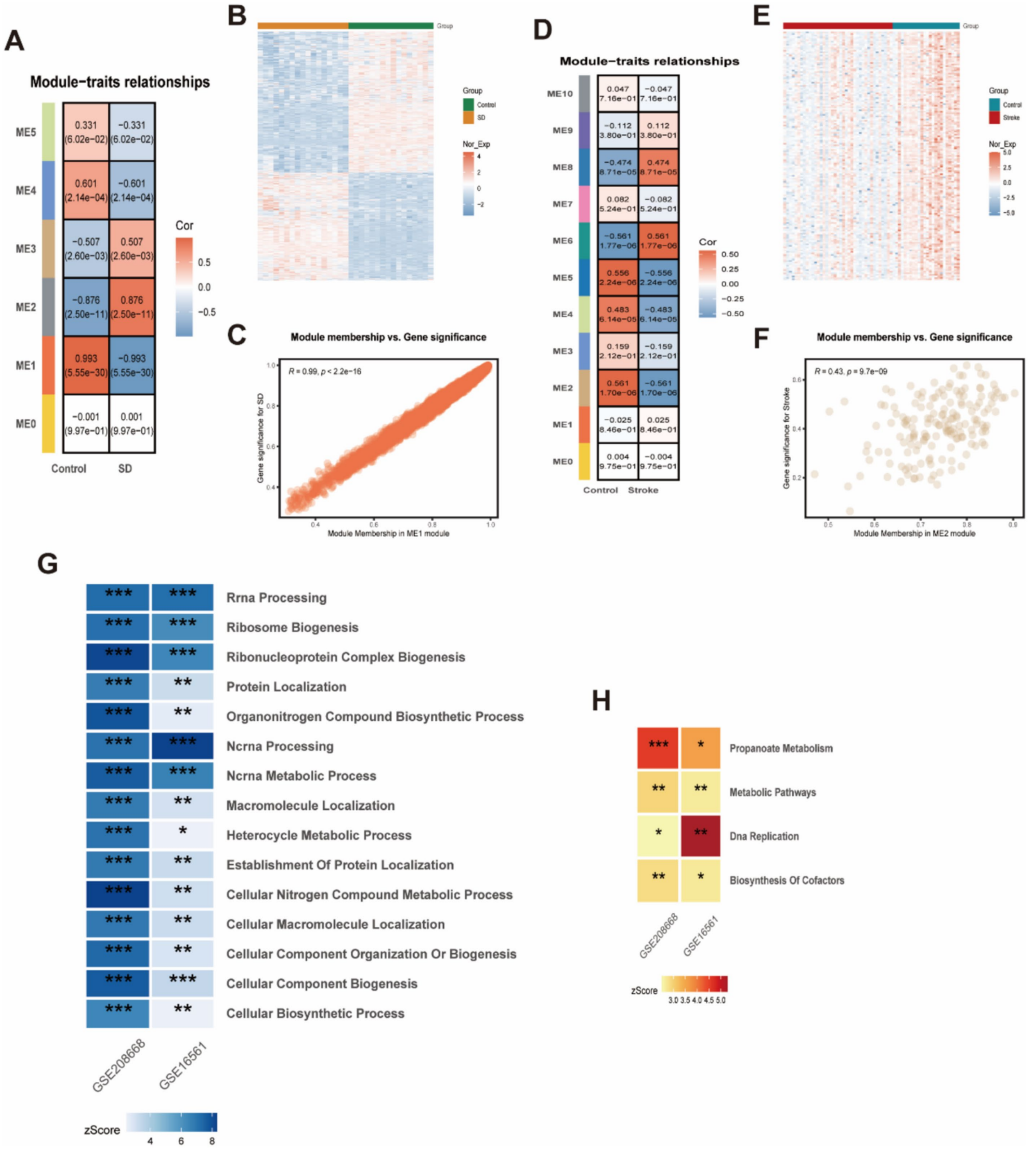
WCGNA analysis of stroke and sleep disorders. **(A,C,D,F)** Heatmap of correlation analysis between genes characterizing the module and clinical phenotypes in patients with stroke and sleep disorders. Red color indicates positive correlation and blue color indicates negative correlation. **(B,E)** Heatmap of Stroke and Sleep Disorder Genes. **(G)** GO enrichment analysis for stroke and sleep disorders. **(H)** KEGG enrichment analysis for stroke and sleep disorders.

**Figure 5:**
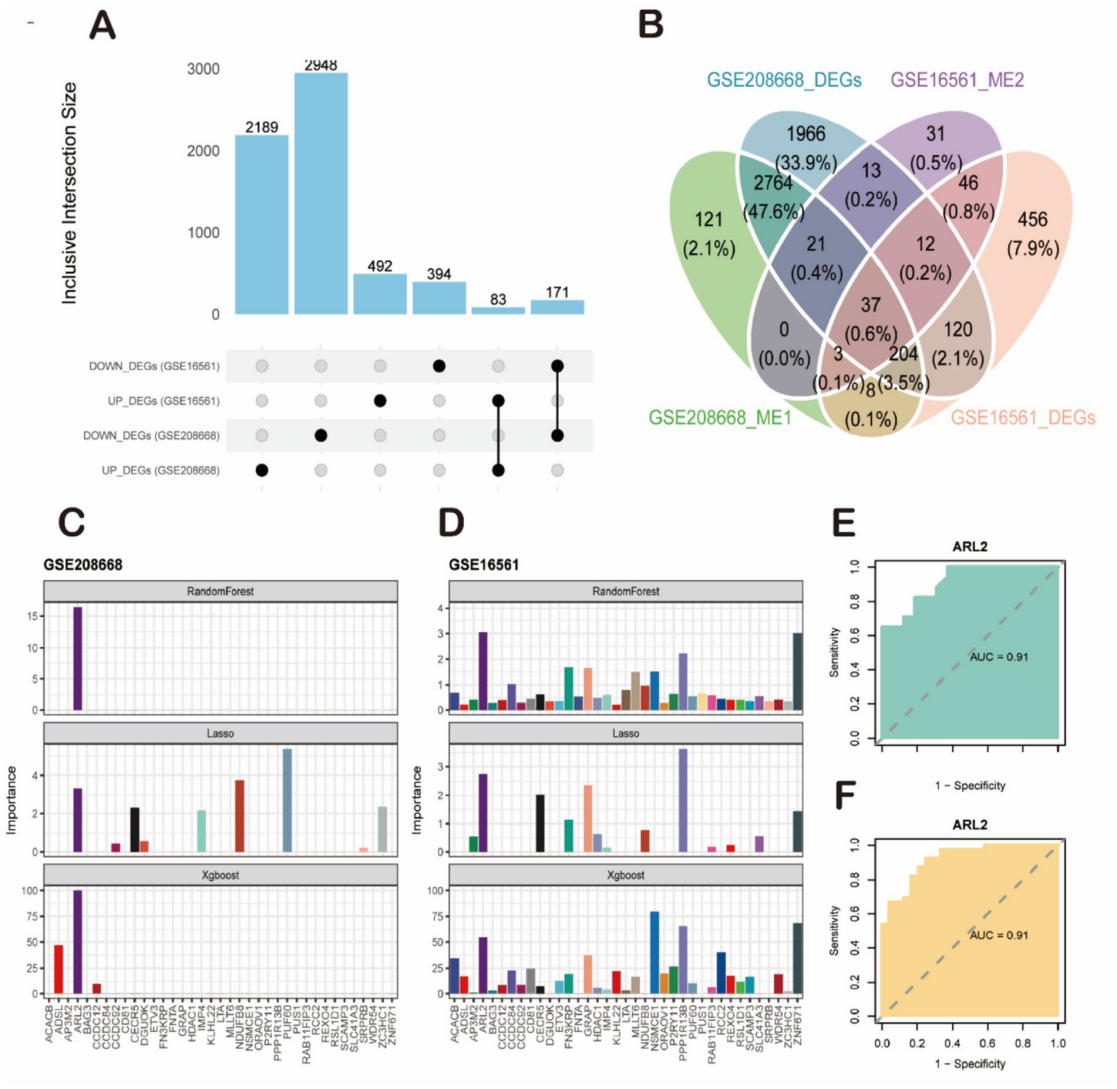
Screening of key candidate genes and validation of their diagnostic efficacy. **(A)** Bar plot showing the number of differentially expressed genes (DEGs) identified from the sleep disorder (SD, GSE208668) and ischemic stroke (IS, GSE16561) datasets. **(B)** Venn diagram illustrating the intersection of four gene sets: GSE208668-DEGs, GSE16561-DEGs, GSE208668_ME1 genes, and GSE16561_ME2 genes, yielding 37 high-confidence candidate genes. **(C,D)** Feature importance analysis of candidate genes using machine learning algorithms. **(C)** Results from the SD dataset (GSE208668). **(D)** Results from the IS dataset (GSE16561). The ARL2 gene was consistently identified as the top-ranked feature by all three algorithms (RandomForest, LASSO, and XGBoost). **(E,F)** Validation of the diagnostic performance of ARL2 using ROC curve analysis. **(E)** ROC curve for ARL2 in classifying SD samples from controls in the GSE208668 cohort (AUC = 0.91, 95% CI: 0.8166–1). **(F)** ROC curve for ARL2 in classifying IS samples from controls in the GSE16561 cohort (AUC = 0.91, 95% CI: 0.8467–0.9824).

We conducted Weighted Gene Co-expression Network Analysis (WGCNA) on two datasets: GSE208668 for SD and GSE16561 for stroke, aiming to investigate the relationship between clinical characteristics and gene expression. After clustering modules based on their similarity, we identified 6 modules in the SD dataset and 11 in the stroke dataset (Figures 4A,D). Correlations between the modules and clinical traits were calculated, revealing that the red module exhibited the strongest positive correlation with SD (*r* = 0.99) (Figure 4C), while the brown module showed the highest correlation with stroke (*r* = 0.56) (Figure 4F). Additionally, gene significance (GS) within the modules was strongly correlated with module membership (MM), with correlations of 0.99 for SD and 0.43 for stroke. This further supports the significant relationship between the module genes and the respective diseases. In total, we identified 61 overlapping genes that may play a pivotal role in the pathogenesis of SD and stroke, as determined by WGCNA. Subsequently, we further took the intersection of these 61 intersecting genes with the 254 common differentially expressed genes obtained previously, and finally identified these 37 high-confidence candidate genes (Figure 5B).

### 3.5 Identification and validation of ARL2 as a key diagnostic gene for SD and stroke using machine learning

To further identify key genes with maximal diagnostic utility, we performed sequential feature selection using three distinct machine learning algorithms: Lasso regression, XGBoost, and Random Forest (Figures 5C,D). This tri-modal approach was applied to the 37 candidate genes, with the Benjamini-Hochberg false discovery rate (FDR) correction implemented during screening to control for multiplicity across all candidate features. The analyses were conducted on two datasets: GSE208668 and GSE16561. Each dataset presents the importance of different genes using the three machine learning models (RandomForest, Lasso, and XGBoost). Lasso regression, which tends to shrink less important feature coefficients to zero, identified a more specific set of genes compared to RandomForest. It provided a more focused list of relevant genes, showing that only a few variables (such as ARL2 and others in both datasets) were significant, with most genes showing low importance. XGBoost also pointed to ARL2 as a key gene, but unlike Lasso, it integrated both linear and non-linear relationships between features, thus identifying additional genes with varying importance, some showing moderate influence in both datasets. For both datasets, RandomForest clearly identified a few top genes as most important, with a strong emphasis on ARL2, as seen in the dataset importance plots. This method highlighted several genes with varying levels of importance, with some showing high relevance to the prediction of sleep disorders or stroke. The AUC (Area Under the Curve) values for ARL2 were reported to be 0.91 in both datasets. This suggests that ARL2 is a highly predictive gene for the conditions studied, with a strong balance between sensitivity (true positive rate) and specificity (true negative rate) based on the ROC curve analyses. To prospectively validate its clinical utility, we performed external validation in two independent cohorts. In the GSE22255 cohort (Control vs. IS), ARL2 achieved an AUC of 0.80 (95% CI: 0.647–0.956; DeLong method). Replication in the GSE98566 cohort (Control vs. SD) yielded stronger discrimination with an AUC of 0.92 (95% CI: 0.8009–1; DeLong method). These results demonstrate the robust and generalizable diagnostic utility of ARL2 across diverse populations (Figures 5E,F).

### 3.6 Expected results of animal experimental models

**Figure 6:**
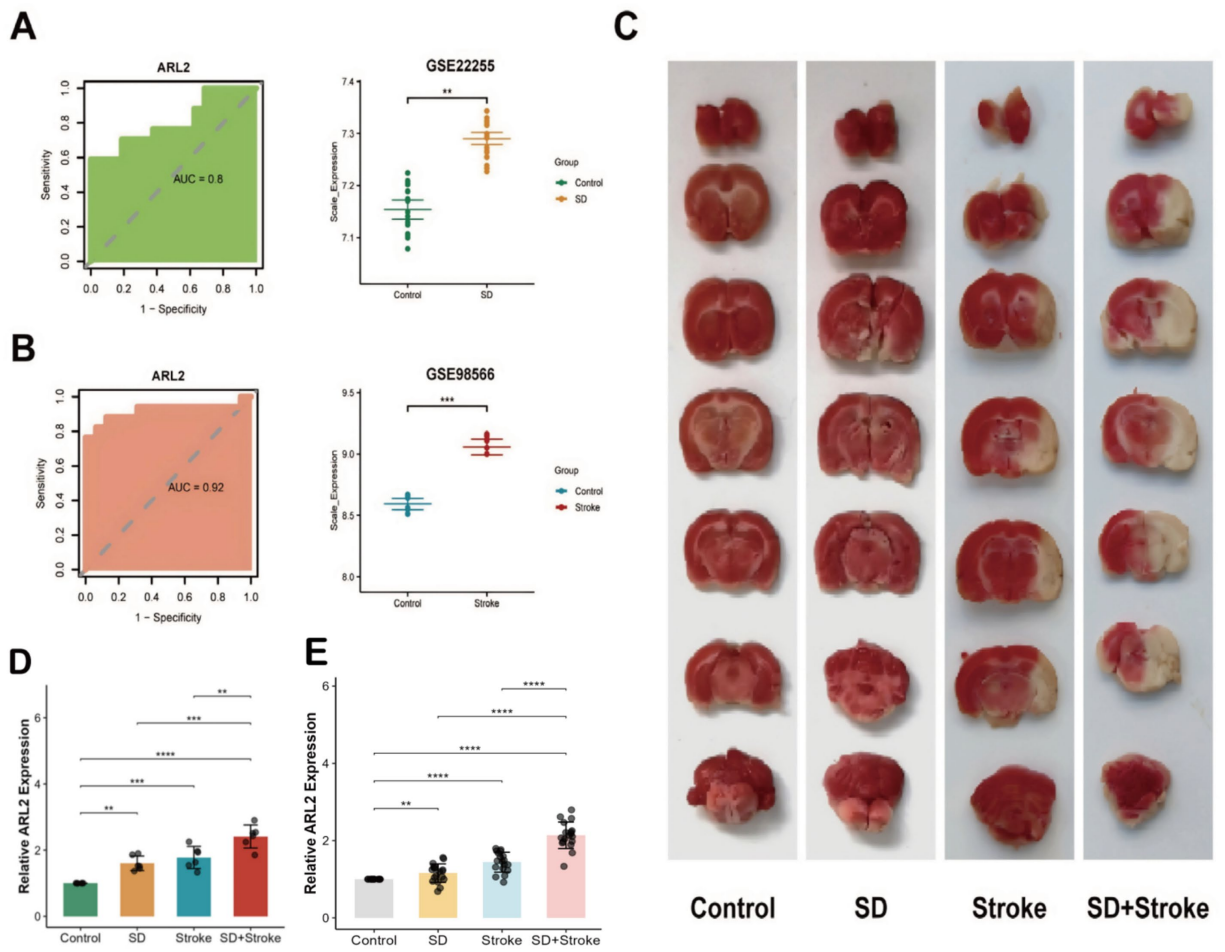
**(A)** ROC curve analysis of ARL2 gene in dataset GSE22255 and expression in control and stroke groups [AUC = 0.80, 95% CI: 0.647–0.956 (DeLong)]. **(B)** ROC curve analysis of ARL2 gene expression in dataset GSE98566 and in control and SD groups [AUC = 0.92, 95% CI: 0.8009–1 (DeLong)]. **(C)** Brain tissue changes in mice in 4 groups. **(D)** Expression of ARL2 gene in mouse brain tissue in group 4. Data are presented as mean ± SD (Standard Deviation). **(E)** Relative expression of ARL2 in peripheral blood from Control, Stroke, SD, and SD + Stroke groups (*n* = 18 per group). Data are expressed as mean ± SD. *p* < 0.05, **p < 0.01, ***p < 0.001 versus the Control group; ###*p* < 0.001 compared to all other groups (one-way ANOVA followed by Tukey’s *post hoc* test).

First, we confirmed that the expression of the internal reference gene *β*-actin was stable (M < 0.5) in all experimental groups by geNorm analysis, as detailed in the Methods section. The relative quantitative results calculated using the 2^(-ΔΔCt) method are shown in Figure 6D. The expression level of 1 gene in the tissue was detected by RT-qPCR. The mRNA expression of ARL2 gene in groups 2, 3 and 4 was significantly higher than that in the normal group. Group 1 (control): baseline expression of ARL2 gene. Group 2 (sleep disorder): ARL2 gene expression was significantly higher than the normal group (Figure 6B). Group 3 (Stroke): Increased expression of the ARL2 gene (Figure 6A). Group 4 (SD + IS): ARL2 expression was increased compared to both groups 2 and 3 (Figure 6D). This may be due to the combined effect of the two conditions. In this case, Group 1 and Group 2, Group 1 and Group 4, and Group 2 and Group 4 were all more significant, while Group 1 and Group 3, and Group 3 and Group 4 were less significant than the former. The brain changes of the four groups of mice are shown in Figure 6C.

### 3.7 Validation of ARL2 expression in an independent human cohort

To advance the translational potential of our findings, we validated the diagnostic value of ARL2 as a circulating biomarker in an independent retrospective human cohort. As shown in Figure 6E, qRT-PCR analysis revealed that the relative expression levels of ARL2 were significantly up-regulated in the Stroke group and the SD group compared to the Control group (*p* = 0.0000198 and *p* = 0.00121, respectively). Most importantly, the expression level of ARL2 in the SD + Stroke group exhibited the most pronounced increase, showing statistically significant differences compared to all other three groups (all *p* < 0.001). These results not only successfully validate our core findings from animal models in human patient samples but also strongly suggest that circulating ARL2 levels could serve as a novel, non-invasive blood-based diagnostic biomarker for distinguishing these disease states.

### 3.8 Exploratory analysis of ARL2-related pathways

To further assess the biological plausibility of ARL2 as a circulating diagnostic biomarker and to explore its potential functional significance in the comorbidity of SD and IS, we performed exploratory co-expression analyses. We investigated the correlation between ARL2 expression and genes comprising two of the important KEGG-shared pathways previously identified (propionate metabolism and oxidative phosphorylation). Interestingly, in the SD dataset GSE208668, we found that ARL2 expression was significantly correlated (FDR < 0.05) with a significant proportion of genes in both pathways, with ARL2 being significantly correlated with 43.8% (28 of 64 genes) in the propionate metabolism pathway, while in the oxidative phosphorylation pathway, it was significantly correlated with 42% (116 of 276 genes) (see Supplementary Table 3 for details). This not only strengthens the credibility of ARL2 as a circulating diagnostic biomarker, but also suggests that its role in SD-IS comorbidities may be functionally linked to dysregulation of these key metabolic processes.

## 4 Discussion

Sleep disturbances are frequently observed in stroke patients and may contribute to an increased risk of stroke, suggesting a bidirectional relationship. Sleep disorders are generally recognized as an independent risk factor for stroke (40). This suggests that one disease may co-exist with another. Shared biomarkers and genetic susceptibility may be a common pathogenic mechanism for haemorrhagic stroke and sleep disorders. Reductions in the risk alleles for sleep disorders PER1 and NR1D1 were found to have a significant impact on the risk of haemorrhagic stroke in one study (41). Our study adds to this body of evidence by identifying common molecular pathways. In addition, the differential diagnosis of sleep disorders combined with idiopathic haemorrhagic stroke is challenging because the clinical manifestations of the two disorders intersect. Patients with haemorrhagic stroke are often associated with severe malaise, excessive daytime sleepiness, and sleep disorders, which may be due to the coexistence of sleep disorders in the patient; However, it is necessary to exclude brainstem injury due to haemorrhagic stroke, sleep apnoea or central nervous system depression (42). Neurocognitive symptoms are also commonly associated with sleep disorders, such as memory loss and attention deficits, which can complicate the clinical differential diagnosis. Therefore, identifying common biomarkers and pathogenic mechanisms is essential for the diagnosis and treatment of these disorders.

Firstly, 254 overlapping differentially expressed genes (DEGs) were identified between the SD and IS datasets, highlighting common genetic factors that may influence both diseases. This set of genes highlights the importance of exploring common molecular pathways to better understand the pathogenesis of SD and IS. By applying WGCNA, six co-expression modules were identified in the SD dataset and 11 co-expression modules in the IS dataset, some of which were strongly correlated with clinical features. The overlapping genes identified in the two datasets are particularly interesting as they suggest a possible common molecular basis between SD and IS. These genes deserve further investigation as potential biomarkers or therapeutic targets for the complications of both diseases. We then put GSE208668 DEGs, GSE208668 ME2, GSE16561 DEGs and GSE16561 ME2 together in a series of machine learning to obtain their common genes, demonstrating how these genes are not only common markers for both sleep disorders and stroke, but may also have a role in both diseases (Figure 3D).

Enrichment analyses revealed several key biological processes and metabolic pathways involved in the common pathogenesis of SD and IS. The enrichment of biological processes such as ‘response to external stimuli’ and ‘organic metabolic processes’ emphasized the impact of external factors, including stress, environmental factors and circadian rhythm disruption, on SD and IS. In addition, metabolic pathways, particularly ‘propionate metabolism’ and ‘oxidative phosphorylation’, were also significantly enriched, suggesting that they may be involved in regulating the sleep–wake cycle and cerebral haemodynamics. In addition, in terms of metabolic pathways and cellular behavior, especially “propionate metabolism” and “oxidative phosphorylation,” suggesting that they may be involved in regulating sleep–wake cycles and cerebral hemodynamics. These findings suggest that metabolic abnormalities and cellular energy dysregulation may be key contributors to both conditions, providing potential avenues for therapeutic intervention. Statistics show that about 50% of IS patients may face gastrointestinal complications (43). Recent studies have shown that ischemic brain injury is related to systemic stress responses associated with activation of ANS sympathetic branches, leading to the release of catecholamines, and long-term stress responses may lead to problems such as gastrointestinal dyskinesia (44). In addition, the microbiota in the intestinal tract of patients with ischemic brain injury has also undergone tremendous changes (45). The study found that the intestinal microorganisms phosphatidylcholine and L-carnitine were converted to trimethylamine, which was further converted into trimethylamine-N-oxide (TAMO) that promotes atherosclerosis by promoting the formation of foam cells by macrophages (46, 47). Intestinal microorganisms produce a series of metabolites including short-chain fatty acids (butyrate, propionate), where propionate is produced mainly by Bacteroidetes, Firmicutes and Ackermanns are produced through the propylene glycol pathway and the acrylate pathway (48, 49). Strokes can lead to homeostasis of intestinal flora and altered short-chain fatty acid metabolism (50). We found that upregulating microbiota diversity and intestinal probiotic abundance, accelerating short-chain fatty acid metabolism, regulating amino acid and energy metabolism, thereby significantly inhibiting the inflammatory cascade and alleviating ischemic brain damage (51). Studies have shown that the increased concentration of short-chain fatty acids (SCFAs) in the feces of elderly patients with insomnia mainly reflects intestinal absorption dysfunction rather than simply increased production. These unabsorbed SCFAs worsen sleep through multiple pathways along the gut-brain axis: First, propionate and other substances activate FFAR3 receptors in the portal vein system, promoting the release of norepinephrine, directly exacerbating physiological hyperarousal and leading to difficulty falling asleep; second, malabsorption leads to insufficient energy in colon cells, damages the intestinal barrier, and triggers systemic low-grade inflammation, which in turn interferes with the sleep center; third, SCFAs further disrupt the sleep–wake rhythm by affecting serotonin synthesis and GABA/glutamate balance. At the same time, a higher BMI, malabsorption of SCFAs, and chronic inflammation form a vicious cycle, which together explain the worse sleep continuity in the short-sleep insomnia phenotype (52). Moreover, people with insomnia tend to have lower gut microbial diversity, a higher ratio of Firmicutes to Bacteroidetes, and lower levels of bacteria that produce short-chain fatty acids (SCFAs) (53). In addition to the gut flora, propionate is also associated with inflammation and oxidative stress, which has anti-inflammatory properties that may reduce the risk of stroke by reducing vascular inflammation. A report pointed out that short-chain fatty acids, including propionate, may regulate recovery after stroke by affecting microglia activation and neuroplasticity (54). Furthermore, studies have found that stroke and insomnia can be linked through the brain-gut axis. Due to brain damage to areas that control circadian rhythms, stroke disrupts sleep regulation, leading to insomnia, which affects up to 40% of stroke survivors. In turn, insomnia exacerbates stroke recovery by increasing inflammation and impairing neuroplasticity. The brain-gut axis plays a crucial role, as stroke-induced dysbiosis in the gut microbiome alters the production of neurotransmitters, such as serotonin, which regulate sleep and mood, further exacerbating insomnia. Conversely, sleep deprivation disrupts the composition of the gut microbiome, increasing systemic inflammation and potentially worsening stroke outcomes, highlighting a vicious cycle in which each condition amplifies the other through neuroinflammatory and microbial pathways (55). Cell respiration is the process by which cells generate energy through mitochondrial oxidation and phosphorylation. During ischemic stroke, reduced blood flow limits oxygen availability, impairs mitochondrial function and turns cells to anaerobic respiration. This produces lactic acid, leading to acidosis and further cellular damage. Reperfusion paradoxically increases the production of reactive oxygen species (ROS), leading to mitochondrial dysfunction and neuronal apoptosis. Studies have shown that excessive ROS caused by impaired cellular respiration can lead to damage to the blood–brain barrier and worsen stroke results (56). Insomnia, characterized by difficulty falling or staying asleep, is associated with disruptions in oxidative phosphorylation, a cellular process in mitochondria that produces ATP. Research has shown that sleep deprivation caused by insomnia impairs mitochondrial function, reduces oxidative phosphorylation efficiency, and leads to reduced ATP production. This disruption increases oxidative stress due to the accumulation of reactive oxygen species (ROS), which damages cellular components and exacerbates sleep disturbances, creating a vicious cycle. Conversely, inefficient oxidative phosphorylation, commonly seen in conditions such as chronic fatigue syndrome and neurodegenerative diseases, may alter energy metabolism in brain regions that regulate sleep, leading to insomnia. Therefore, the interplay between insomnia and oxidative phosphorylation highlights a bidirectional relationship in which sleep diorders and mitochondrial dysfunction reinforce each other, impacting overall health (57, 58).

In our study, we analyzed these DEG genes by GO and KEGG enrichment with modular genes. We found that propionate metabolism and metabolic pathways featured prominently in the analysis of common drivers of sleep disorders and stroke, with ‘propionate metabolism’ being significantly enriched. Then through machine learning we identified ARL2 as a prominent gene with high diagnostic value, and its robustness as a predictive biomarker further emphasizes its role in the pathophysiology of SD and IS. By integrating single-cell transcriptomic data, a more nuanced understanding of how specific neuronal subtypes and immune cells contribute to the shared pathophysiology of SD and IS is possible. This is particularly important as it highlights the importance of cellular stress responses, inflammation and circadian rhythms, which are common in both diseases.

ARL2 is a member of the ARF family and the RAS superfamily of regulatory GTPases (59), which are highly conserved and commonly expressed in eukaryotes (60). It plays a role in the regulation of microtubule protein folding and microtubule disruption (61, 62) and is present in cytoplasmic lysates tightly bound to the microtubule protein-specific co-chaperone cofactor D, which shares these activities. Numerous studies have shown that cerebral ischemia leads to mitochondrial dysfunction, ATP depletion, cytoskeletal destruction, and ultimately necrosis or apoptosis (63). Whereas, Arl2 is implicated in mitochondrial function, such as mitochondrial morphology, motility, and maintenance of ATP levels (64). And in a similar study of cardiomyocytes, miR-15b downregulates and regulates cellular ATP levels via Arl2 (65). In the prevention of cerebral ischemia–reperfusion injury, miR-15b has also been shown to inhibit the expression of ADP ribosylation factor-like 2 and reduce the level of adenosine triphosphate in mice treated with mild hypothermia in mice with middle cerebral artery occlusion (66). These findings underscore an association between ARL2 and stroke. Because ARL2 regulates mitochondrial morphology, motility, and ATP levels, it can indirectly affect metabolic pathways occurring in mitochondria, such as propionate metabolism. We infer that disruption of mitochondrial integrity may affect the efficiency of enzymes involved in propionate metabolism. On the other hand, the related protein ADP-ribosylation factor-associated protein 1 (ARFRP1) is involved in lipid droplet (LD) growth and lipolysis, and is associated with lipid metabolism during the transmission of hepatitis C virus. Although ARFRP1 and ARL2 are different, their shared membership in the ARF family suggests that ARL2 may also interact with lipid-related pathways, and propionate is a short-chain fatty acid (67). We further infer that ARL2 is associated with propionate metabolism. The circadian rhythm has a complex bidirectional relationship with mitochondrial function. The chromatin immunoprecipitation (ChIP) sequencing data set of Bmal1, Clock and Cry shows that various functions of mitochondria are under the control of circadian rhythm (68, 69). In addition, post-transcriptional mechanisms (e.g., protein acetylation) are also involved in the circadian rhythm of mitochondria (70). The clock controls the expression/activity of fission proteins (such as Drp1, Fis1) and autophagy-related proteins (such as Bnip3, Pink1), driving the diurnal cycle of mitochondrial fusion and cleavage. Deletion of clock genes can also disrupt morphological rhythms and functions. Mitochondrial feedback also regulates the circadian rhythm through the NAD + -SIRT pathway and the AMPK energy sensing pathway. Clock-driven NAD + oscillation not only affects mitochondria (SIRT3), but also affects the activity of SIRT1 in the nuclear nucleus. SIRT1 rhythmically binds to the CLOCK: BMAL1 complex, deacetylation and promotes PER2 degradation, and enhances clock gene transcription amplitude. SIRT1 also acts as a histone deacetylase, interacts with CLOCK to participate in chromatin remodeling and regulates clock gene expression (71). In addition, the ATP rhythm generated by Drp1-mediated changes in mitochondrial morphology itself can directly feedback affecting the core clock oscillator (72). In this study, we quantified ARL2 mRNA expression using RT-qPCR. Results revealed that the expression of these genes was significantly altered in the experimental group compared to the control group. Specifically, genes associated with inflammatory responses and cellular stress, such as ARL2, showed increased expression in the SD, IS, and SD + IS groups, with the most pronounced changes in the SD + IS group. This suggests that the combination of sleep disorders and ischaemic stroke may have a superimposed or synergistic effect on gene expression, especially genes associated with inflammation, oxidative stress and neuronal damage. ARL2 was significantly upregulated in all cases, especially in the SD + IS group, suggesting that ARL2 may be a potential biomarker for SD and IS. These findings support the hypothesis that common molecular pathways, including inflammation and metabolism, underlie both conditions, thus providing insights into their common pathophysiology.

Furthermore, we validated the differential expression of ARL2 in an independent retrospective human cohort. The consistent upregulation of ARL2 in the blood of patients with stroke, sleep disorder, and most notably in those with both conditions, strongly corroborates our pre-clinical findings and underscores the translational relevance of ARL2 as a peripheral biomarker. Notably, this clinical validation aligns with the previously discussed involvement of ARL2 in mitochondrial function and neuroinflammatory pathways, providing a mechanistic basis for its upregulation under conditions of hypoxic and inflammatory stress, such as those characterizing OSA and stroke comorbidity. Although the comprehensive assessment and diagnosis of clinical sleep disorders typically involve a multi-faceted approach, our study utilized the well-validated Pittsburgh Sleep Quality Index (PSQI, provided in Supplementary Table 5) to define sleep disorder, thereby ensuring standardized and reproducible cohort criteria.

While this study provides compelling evidence, future prospective studies with larger sample sizes and more detailed clinical profiles are warranted to further establish the diagnostic and prognostic utility of ARL2. Nevertheless, our data identify circulating ARL2 as a promising and readily measurable biomarker that could facilitate risk stratification and early detection of stroke, especially among individuals with pre-existing sleep disturbances. The RT-qPCR results reinforce the concept that SD and IS share overlapping molecular mechanisms, particularly in the regulation of inflammation and stress-response genes. These insights may guide future therapeutic strategies targeting common pathways to concurrently treat both conditions. Additional studies are needed to validate these findings and explore the functional roles of these genes in disease progression.

Building upon these clinical findings, it is important to understand the molecular context of ARL2. From the above, we know that ARL2 is part of the broader ADP-ribosylation factor (ARF) family, which acts as a cofactor in the ADP-ribosylation process. ADP-ribosylation, catalyzed by enzymes such as PARP1 and tankyrases, regulates inflammatory responses through pathways involved in tumor necrosis factor (TNF)-induced cell death and immune signaling (73). While Aβ peptides of 39–42 amino acids are the primary component of protein aggregates found in senile plaques of Alzheimer’s disease and impaired mitochondrial electron transport along with exposure to metal ions can lead to Aβ aggregation and subsequent activation of PARP1, which is closely linked to several neurological diseases (74), our focus remains on ARL2’s potential influence on neuroinflammatory processes in brain regions such as the cerebral cortex and hippocampus in the context of sleep disorders and stroke.

In summary, this study explored and identified for the first time the pivotal genes of sleep disorders and stroke, and analyzed the possible pathogenesis. Unlike many studies that focus on tissue-specific biomarkers or single pathological pathways, we purposely utilized circulating transcriptome data as well as an integrated computational biology approach combining WGCNA and multiple machine learning algorithms. Through this strategy, we identified ARL2 as a novel circulating biomarker for the detection of the comorbidity of SD and IS, which gives our study a significant clinical advantage for non-invasive, rapid screening, identification of people at high risk for sleep disorders, and prevention of stroke. Besides, going beyond single-disease inflammatory biomarker studies, our data-driven approach uniquely reveals the relationship between ARL2 and dysregulation of mitochondrial metabolic pathways (propionate metabolism and oxidative phosphorylation) in both SD and IS, which reveals a previously underappreciated pathogenic axis and opens up the possibility of new treatments beyond common anti-inflammatory and antioxidant strategies. However, this paper also has some limitations. Further functional studies are needed to validate the specific role of the ARL2 gene in SD and stroke and to explore its functional mechanisms in disease progression.

Despite the encouraging findings, this study has several limitations. First, while our integrated bioinformatics and machine learning approach strongly identified ARL2 as a common diagnostic biomarker, the precise causal relationship between ARL2 dysregulation and the pathogenesis of sleep disorders (SD) and ischemic stroke (IS) remains to be fully elucidated. The observed association, while strong, does not confirm causality. Second, our analysis relied primarily on publicly available transcriptomic datasets and a single validation cohort. The relatively small sample size, particularly in the SD dataset, and the retrospective nature of the clinical validation warrant confirmation in larger, prospective, multicenter studies to increase the generalizability of our findings. Finally, while we validated ARL2 expression in animal models and human blood samples, the underlying functional role of ARL2 in the coexisting mechanisms of SD and IS has not been experimentally validated. Future studies employing gene knockout or overexpression in relevant animal models will be crucial to uncover the underlying molecular pathways.

## Data Availability

The original contributions presented in the study are included in the article/Supplementary material, further inquiries can be directed to the corresponding author/s.

## Footnotes

^1^ http://www.ncbi.nlm.nih.gov/geo
