# Supplementary figures and images for: Exploring common circulating diagnostic biomarkers for sleep disorders and stroke based on machine learning
Source: Front Neurol. 2025 Oct 30;16:1599135. doi: 10.3389/fneur.2025.1599135 (PMC12611825; doi:10.3389/fneur.2025.1599135)

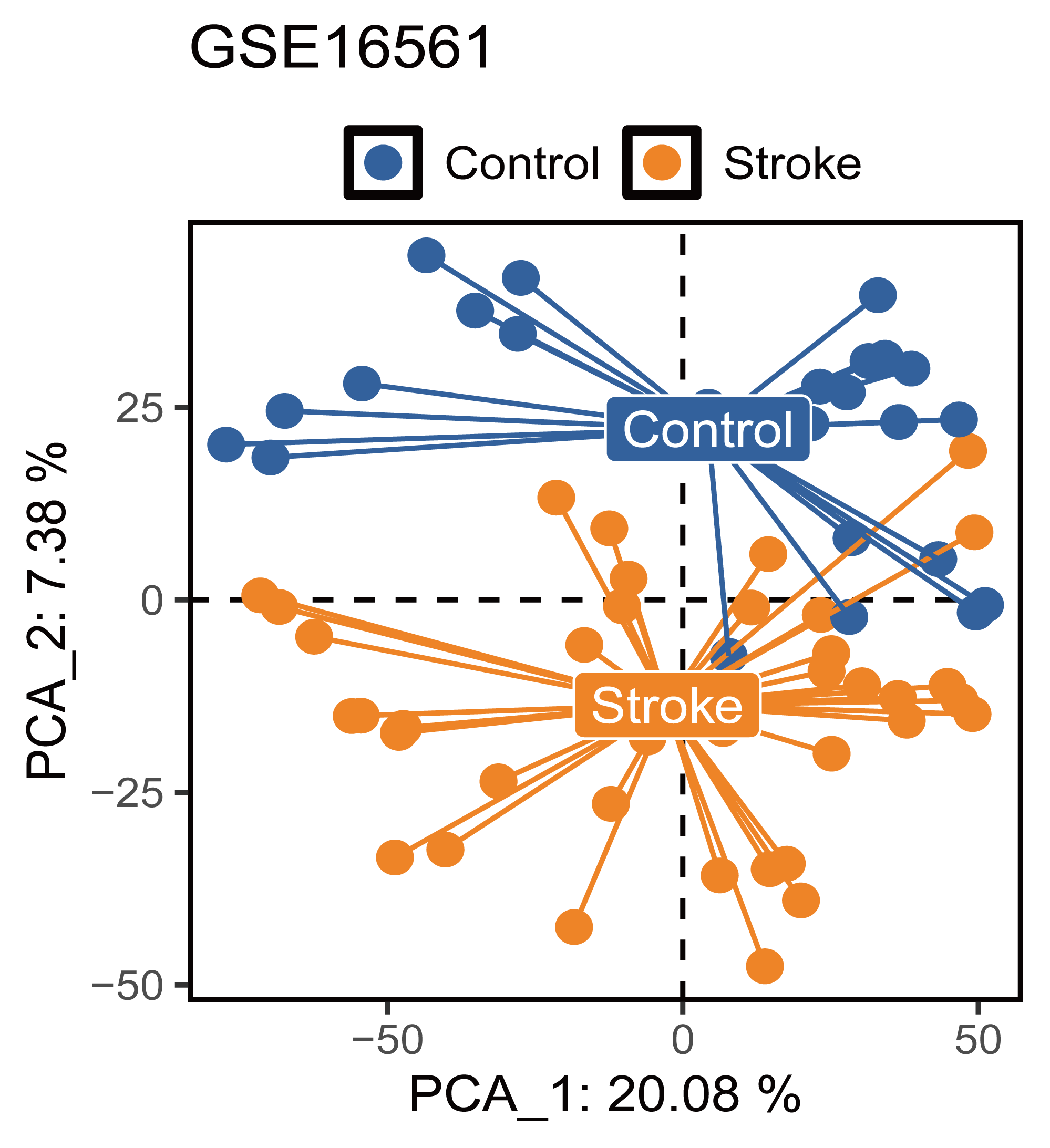

Supplement: Supplementary file 7 [file Image_1.TIF]

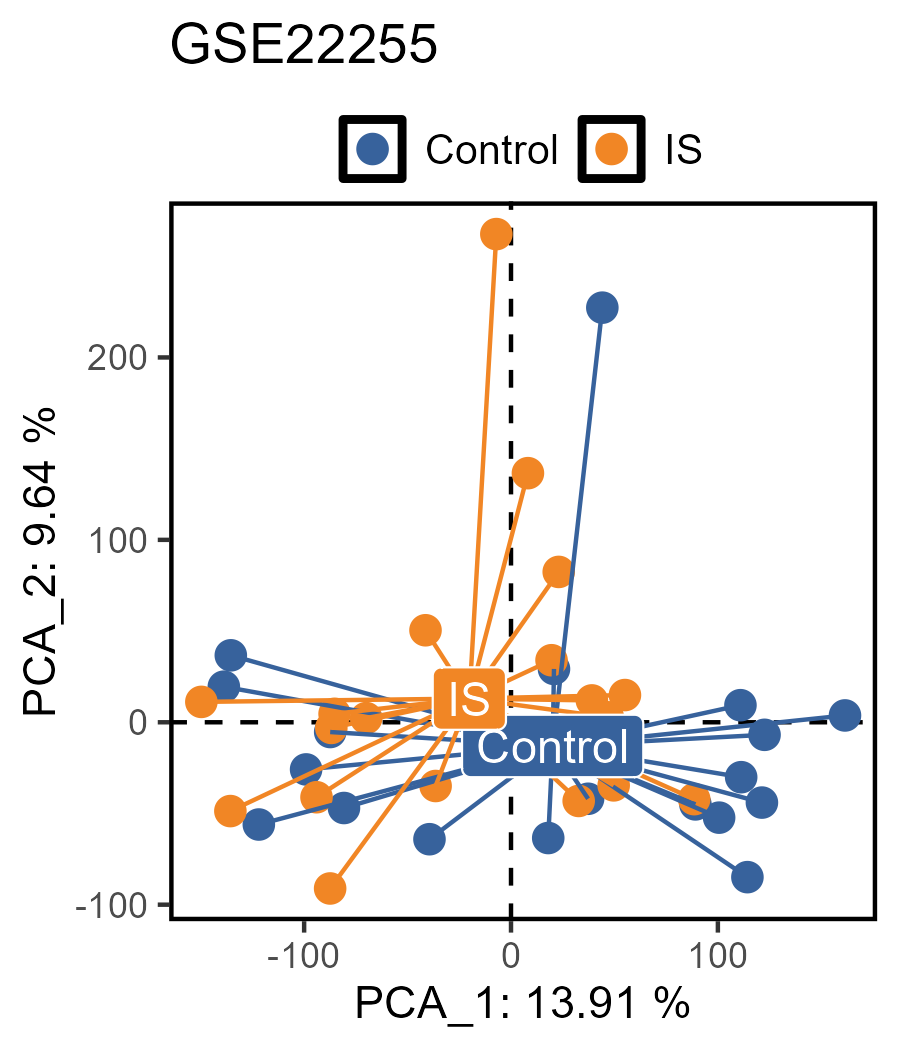

Supplement: Supplementary file 8 [file Image_2.TIFF]

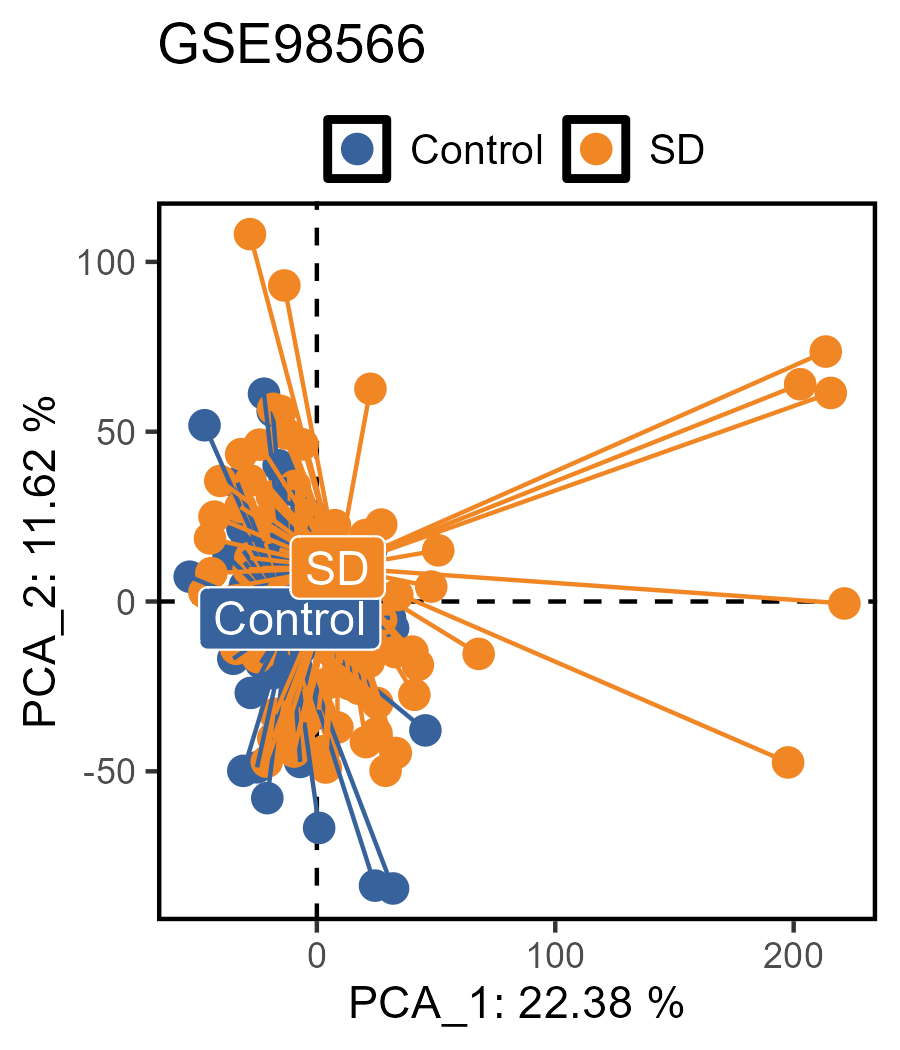

Supplement: Supplementary file 9 [file Image_3.TIFF]

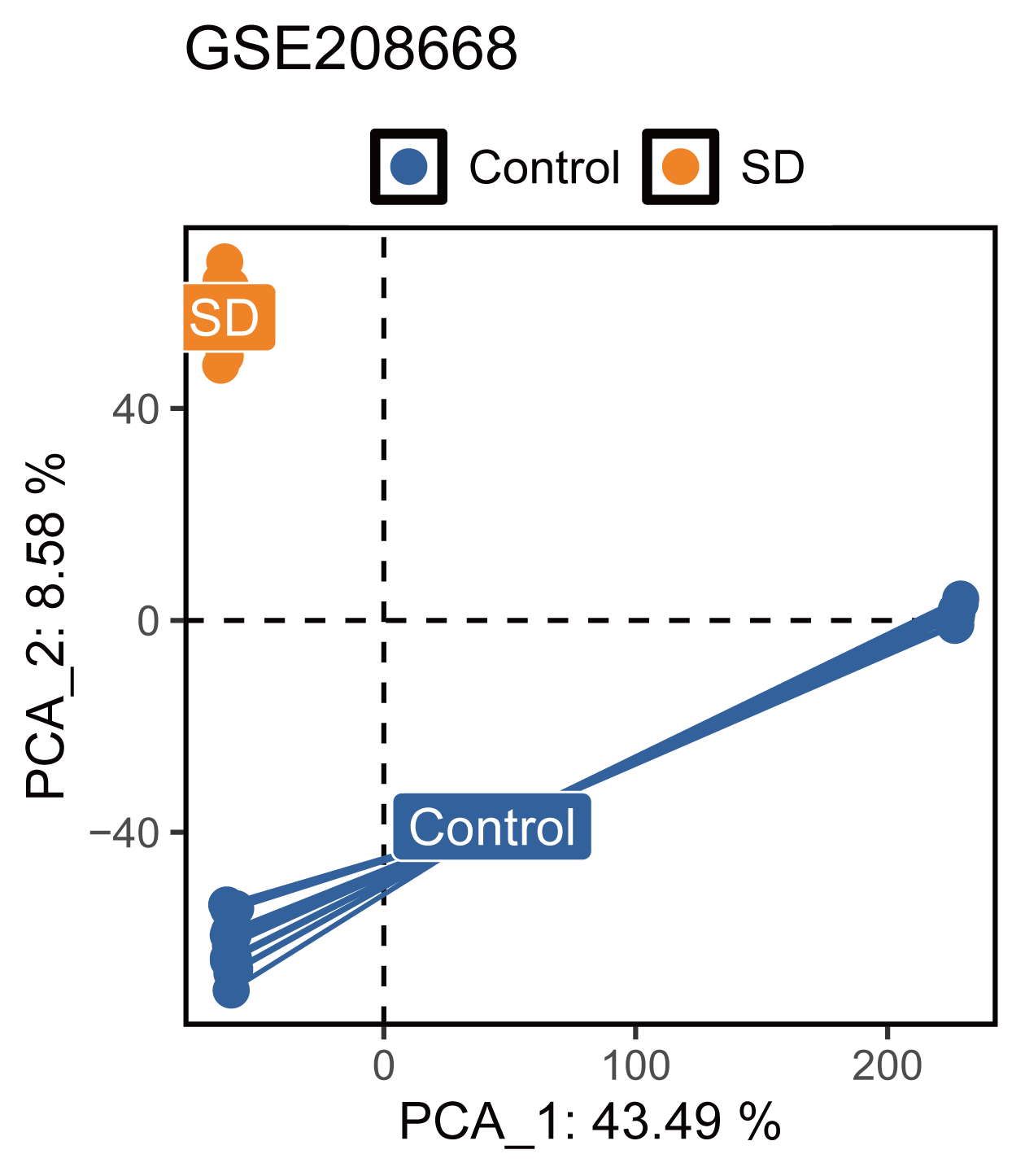

Supplement: Supplementary file 10 [file Image_4.TIF]
